# Development of a Chitosan-Silver Nanocomposite/β-1,3-Glucan/Hyaluronic Acid Composite as an Antimicrobial System for Wound Healing

**DOI:** 10.3390/polym17030350

**Published:** 2025-01-27

**Authors:** Cheng-Jung Yao, Shu-Jyuan Yang, Ming-Jium Shieh, Tai-Horng Young

**Affiliations:** 1 Institute of Biomedical Engineering, College of Medicine and College of Engineering, National Taiwan University, No. 1, Section 1, Jen-Ai Road, Taipei 100, Taiwan; d99548010@gmail.com (C.-J.Y.); image0120@gmail.com (S.-J.Y.); thyoung@ntu.edu.tw (T.-H.Y.); 2 Division of Gastroenterology, Department of Internal Medicine, Wan Fang Hospital, No. 111, Section 3, Xinglong Road, Taipei 116, Taiwan; 3 Department of Oncology, National Taiwan University Hospital and College of Medicine, No. 7, Chung-Shan South Road, Taipei 100, Taiwan

**Keywords:** chitosan, silver nanoparticles, β-1,3-glucan, hyaluronic acid, antibacterial efficiency, blood coagulation, wound healing

## Abstract

An ideal wound dressing should be biocompatible, exhibit high antibacterial activity, and promote blood coagulation in the wound. In this study, we used chitosan as a multifunctional template to synthesize silver nanoparticles embedded in chitosan (Ag NP@CHI), which were then combined with β-1,3-glucan/hyaluronic acid (HA) to form an Ag NP@CHI/β-1,3-glucan/HA composite material with biocompatibility, wound healing-promoting properties, and antibacterial activity. A high concentration of chitosan led to the formation of smaller crystalline structures of Ag NPs and improved their dispersion within the chitosan matrix, but decreased their antibacterial potency. The Ag NP@CHI prepared with 1.0 mg/mL chitosan had the smallest particle size and good antibacterial activity. Compared to Ag NP@CHI, the prepared Ag NP@CHI/β-1,3-glucan/HA composite significantly enhanced biocompatibility, cell migration, hemocompatibility, and blood coagulation, with a minor reduction in antibacterial efficiency due to restricted ionic silver release and diffusion. With its high biocompatibility, hemocompatibility, promotion of blood coagulation and wound healing, and antibacterial efficiency, Ag NP@CHI@β-1,3-glucan/HA demonstrates potential as a wound healing composite in the future.

## 1. Introduction

The wound site provides an ideal environment for microbial growth, and the proliferation of microorganisms at the injured site delays the healing process. Reducing microbial infection is, therefore, crucial for effective wound recovery. Approximately 4% of hospitalized patients are estimated to suffer from serious infections, imposing an economic burden of about US$25 billion annually [1,2]. Traditional wound treatment methods, such as cotton gauze, provide a physical barrier, absorb exudate, and protect against mechanical damage [3]. However, cotton gauze lacks inherent antimicrobial properties. An ideal wound dressing should exhibit high exudate absorption capacity, antibacterial properties, non-cytotoxicity, and excellent biocompatibility [4]. Polysaccharides are considered effective wound-healing materials due to their high biocompatibility, biodegradability, wound repair acceleration, and adaptability to various forms. Consequently, various polysaccharide-based wound dressings have been developed, including those based on alginate, chitosan, carrageenan, pectin, and dextran [5,6,7,8].

Chitosan, a natural functional polymer derived from the deacetylation of chitin, is nonirritating, nontoxic to human tissues, and capable of reducing tissue irritation and inflammation. It exhibits a growth-inhibitory effect on fungi and bacteria such as *Escherichia coli* (*E. coli*) and *Staphylococcus aureus* (*S. aureus*), thereby reducing the risk of wound infection. Additionally, chitosan promotes wound healing by facilitating hemostasis, enhancing the function of inflammatory cells, and improving tissue granulation [9]. It can also serve as a drug carrier, improving drug bioavailability, which benefits local absorption and drug efficacy in wound treatment [6].

Silver nanoparticles (Ag NPs) exhibit remarkable antibacterial properties due to their surface and quantum size effects [10]. They disrupt bacterial membrane structures and inhibit enzyme activity, effectively preventing bacterial drug resistance, a common limitation of antibiotics [11,12]. Consequently, Ag NPs and their complexes are extensively utilized for antibacterial applications and wound healing [13,14]. The preparation of Ag NP-containing complexes falls into three main categories: mixing [15], surface coating [16], and covalent cross-linking [17,18]. However, these methods face challenges, such as difficulty in achieving uniform nanoparticle dispersion and the involvement of complex chemical reactions and purification processes [19]. Additionally, the aggregation of Ag NPs within these complexes can lead to toxicity in injured cells and tissues, limiting their use in wound dressings [20,21].

To address these limitations, in situ reduction of silver nanoparticles is employed, enabling uniform dispersion and preventing aggregation. Research indicates that chitosan serves as an effective multifunctional template, acting as both a reducing agent and a stabilizer during the synthesis of chitosan-silver or chitosan-gold nanocomposites [22,23,24]. The reduction of silver ions (Ag^+^) to metallic silver (Ag^0^) can occur at room temperature and is accelerated by increasing the reaction temperature or chitosan concentration [25]. Furthermore, the concentration of chitosan influences not only the reduction rate but also the nanoparticle size, which directly impacts antibacterial efficacy. Smaller nanocomposite sizes enhance antibacterial performance [26]. Ag NP-chitosan composite materials offer outstanding antibacterial properties, promote wound healing, minimize scar formation, and exhibit excellent biodegradability, reducing the risks associated with prolonged use.

Hyaluronic acid (HA), a key component of the extracellular matrix (ECM), is a natural endogenous polymer that plays a critical role in wound healing and tissue repair due to its unique properties and diverse physiological functions [27]. High-molecular weight HA is essential for tissue hydration, stabilization of the ECM structure, osmotic balance, and anti-inflammatory activity. In contrast, low-molecular weight HA promotes angiogenesis but also exhibits pro-inflammatory activity [28]. These properties allow HA to influence various pathological and physiological processes, including wound healing, inflammation, and angiogenesis. Recent advancements in HA-based wound dressings focus on modifying wound physiology, creating a moist environment, enhancing granulation and epithelialization, and facilitating the delivery of bioactive agents. HA-based hydrogels, with their 3D polymer networks, can absorb significant amounts of water, maintaining a moist environment conducive to cell growth. Their porous structure supports the transport of cells, gasses, and nutrients, making them suitable for addressing scars, asymmetries, and soft tissue defects [29,30]. Hylase Wound Gel^®^, a commercially available hydrogel containing 2.5% sodium hyaluronate, is designed to prevent tissue dehydration and support the healing process.

β-1,3-Glucans, derived from yeast, cereals, and fungi, are glucose polymers classified as biological response modifiers [31]. Both granular and soluble forms of β-d-glucan can enhance immune function and exhibit anti-infectious, antitumor, and immunomodulatory activities [32,33,34,35]. Some β-glucans demonstrate antimicrobial properties against various Gram-positive and Gram-negative bacteria [36]. The immunostimulatory effect of β-glucans on wound healing primarily operates through two mechanisms: indirect activation of macrophages via cytokines and direct effects on keratinocytes and fibroblasts. Activated macrophages release growth factors that support cell proliferation, angiogenesis, epithelial regeneration, and increased wound tensile strength [37].

**Scheme 1:**
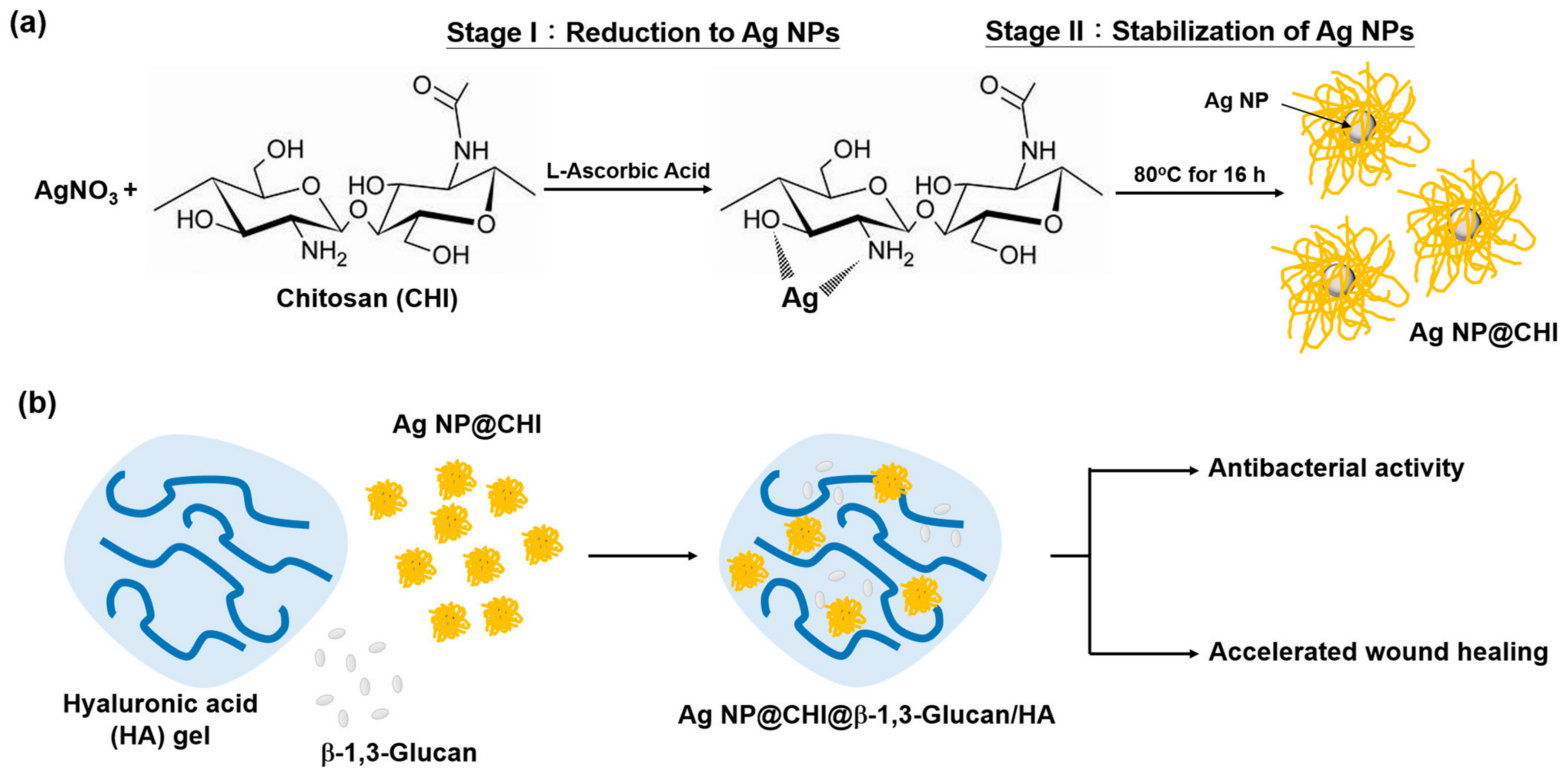
(**a**) Mechanism of the synthesis of silver nanoparticles–chitosan nanocomposites (Ag NP@CHI) using chitosan (CHI) as a template to reduce Ag^+^ ion to Ag^0^ via L-ascorbic acid and as a stabilizer to maintain the Ag NPs in the nanocomposites. (**b**) Synthesis of Ag NP@CHI@β-1,3-glucan/HA for antibacterial activity and wound healing.

In this study, chitosan was used as a template to reduce silver ions (Ag^+^) to metallic silver (Ag^0^) using L-ascorbic acid and as a stabilizer to ensure the uniform dispersion of generated silver nanoparticles (Ag NPs) in this chitosan-silver nanocomposites (Ag NP@CHI). To provide a moist environment at the wound site, enhancing granulation and epithelialization, the Ag NP@CHI was incorporated into β-1,3-glucan/HA-based composite to form Ag NP@CHI@β-1,3-glucan/HA. And the antibacterial activity, cytocompatibility, and cell migration properties of Ag NP@CHI@β-1,3-glucan/HA were evaluated to determine its potential as a wound healing composite (Scheme 1).

## 2. Materials and Methods

### 2.1. Materials

Chitosan with a minimum degree of deacetylation of 85% and molecular weight of 15 kDa was sourced from Polysciences, Inc. (Warrington, PA, USA). Silver nitrate (99.85%) was obtained from Acros Organics (Fair Lawn, NJ, USA). L-ascorbic acid (≥99%) and 3-(4,5-dimethylthiazol-2-yl)-2,5-diphenyltetrazolium bromide (MTT) were purchased from Sigma-Aldrich (St. Louis, MO, USA). HA with a molecular weight of 2000 kDa was procured from Kikkoman Biochemifa Company (Tokyo, Japan). β-1,3-glucan with a molecular weight of 20 kDa was acquired from DISAM Biotechnology CO., LTD. (Taipei, Taiwan), and HealiAid^®^ Collagen Wound Dressing was obtained from Maxigen Biotech Inc. (Taoyuan, Taiwan).

### 2.2. Preparation of Ag NP@CHI

Ag NP@CHI were synthesized using a modified procedure from Canama et al. [38]. In brief, varying volumes of 5 mg/mL AgNO_3_ solution were added to 500 μL of a chitosan solution with concentrations of 0.5, 1, 2.5, or 5 mg/mL and vortexed for 5 s. Subsequently, 200 μL of a 10 mg/mL L-ascorbic acid solution was introduced and vortexed for 20 s to facilitate the reduction of silver ions (Ag^+^) into metallic silver. The mixtures were incubated in an oven at 80 °C for 1 and 2 days before being transferred to amber bottles for subsequent analyses.

### 2.3. Preparation of Ag NP@CHI@β-1,3-Glucan/HA

For the preparation of the β-1,3-glucan/HA composite solution, 27.2 mg of HA powder and 43 mg of β-1,3-glucan were dissolved in 5 mL of deionized water (ddH_2_O). To form the composite (Ag NP@CHI@β-1,3-glucan/HA), 800 μL of Ag NP@CHI, with an Ag concentration of 250 ppm, was drawn into a 5 mL syringe, while 4.2 mL of the β-1,3-glucan/HA composite solution was drawn into another syringe. The two solutions were mixed thoroughly by pumping them back and forth through a three-way stopcock. A control composite without Ag NPs (CHI@β-1,3-glucan/HA) was prepared by mixing 800 μL of chitosan solution with 4.2 mL of the β-1,3-glucan/HA composite solution.

### 2.4. Characterization of Ag NP@CHI and Ag NP@CHI@β-1,3-Glucan/HA

The relationship between particle size and surface charge of Ag NP@CHI was analyzed in relation to the content of AgNO_3_, chitosan concentration, and heating duration at 80 °C, using the Zetasizer Nano-ZS (Malvern Instruments Ltd., Malvern, Worcestershire, UK). The surface morphology of Ag NP@CHI, prepared under varying conditions and precipitated onto 300-mesh carbon-coated copper grids, was examined via transmission electron microscopy (TEM, Hitachi H-7500, Tokyo, Japan). Powder X-ray diffraction (XRD) patterns of chitosan and Ag NP@CHI prepared under different conditions were recorded using a D2 PHASER X-ray diffractometer (Bruker AXS Inc., Fitchburg, WI, USA) with Cu Kα radiation (λ = 1.5418 Å). The FTIR spectra of freeze-dried samples of Ag NP@CHI, Ag NP@CHI@β-1,3-glucan/HA, and CHI@β-1,3-glucan/HA were analyzed using a Nicolet™ Summit FTIR Spectrometer equipped with MicromATR (Thermo Fisher Scientific, Waltham, MA, USA) within the wavelength range of 400–4000 cm^−1^.

### 2.5. Cytotoxicity of Ag NP@CHI and Ag NP@CHI@β-1,3-Glucan/HA

Mouse NIH/Swiss embryo fibroblast cells (NIH/3T3), obtained from the American Type Culture Collection (ATCC), were cultured in Dulbecco’s Modified Eagle’s Medium supplemented with a 10% fetal bovine serum (Gibco, Waltham, MA, USA), 100 U/mL penicillin G sodium salt, 100 μg/mL streptomycin, 250 ng/mL amphotericin B (Biological Industries, Cromwell, CT, USA), and 4.876 g/L sodium bicarbonate (NaHCO_3_). Cells were maintained at 37 °C in a humidified incubator with 5% CO_2_. For sub-culturing, adherent cells were detached using Trypsin-EDTA (Gibco, Waltham, MA, USA), and the medium was replaced every two days.

NIH/3T3 cells (1.5 × 10^4^ per well) were seeded in 96-well plates and incubated for 24 h. After incubation, the medium was replaced with test media containing AgNO_3_, Ag NP@CHI, Ag NP@CHI@β-1,3-glucan/HA, and CHI@β-1,3-glucan/HA at silver concentrations of 3.1, 6.3, 12.5, 25, and 50 ppm. Following 24 h of exposure, the test medium was replaced with a fresh medium containing MTT reagents and incubated for an additional 3 h. Absorbance was then measured at 570 nm using an ELISA microplate reader (SpectraMax M2 Multi-Mode Microplate Reader, Molecular Devices, USA). Cell viability was calculated as a percentage relative to the untreated control group [39].

### 2.6. Hemocompatibility of Ag NP@CHI and Ag NP@CHI@β-1,3-Glucan/HA

Six male Wistar rats (340–360 g) were obtained from BioLASCO Shortcourse, Taiwan. All in vivo experimental procedures were reviewed and approved by the National Taiwan University College of Medicine and the College of Public Health Institutional Animal Care and Use Committee (Approved NO. 20220416, 5 June 2023). During animal experimental studies, all experimental rats received care according to the guidelines outlined in the Guide for the Care and Use of Laboratory Animals (8th edition).

Heparin-stabilized whole blood samples were collected from Wistar rats, washed 3–4 times with Phosphate-buffered saline (PBS) to remove the plasma and the buffy coat, and then diluted with PBS. A 300 μL aliquot of blood was mixed with 300 μL of Ag NP@CHI, Ag NP@CHI@β-1,3-glucan/HA, or CHI@β-1,3-glucan/HA (Ag concentrations serially diluted from 50 to 0.4 ppm), PBS (negative control, NC), or ddH_2_O (positive control, PC). The mixtures were stirred at 100 rpm and incubated at 37 °C for 1 h. Subsequently, the samples were centrifuged at 4000 rpm for 5 min, and the absorbance of the supernatant was measured at 540 nm using an ELISA microplate reader. The hemolysis percentage was calculated using the following Equation (1) [39]:

Hemolysis percentage (%) = (OD_test samples_ − OD_negative control_)/(OD_positive control_ − OD_negative control_) × 100 (1)

### 2.7. Coagulation Time of Blooding (CBT) of Ag NP@CHI@β-1,3-Glucan/HA

For the coagulation assay, 5 mg of Ag NP@CHI, Ag NP@CHI@β-1,3-glucan/HA, CHI@β-1,3-glucan/HA, or β-1,3-glucan/HA was placed in a 1.5 mL Eppendorf tube. Subsequently, 0.5 mL of anticoagulant-treated whole blood (3.8% sodium citrate, blood ratio = 1:9) from Wistar rats was added, followed by 100 μL of 0.1 M CaCl_2_. The tubes were tilted every 20 s to observe the coagulation process. Complete coagulation was defined as the point when the blood stopped flowing upon tilting the tube to 90°. Coagulation time was recorded as CBT. NC consisted of blood and CaCl_2_ only, while HealiAid^®^ Collagen Wound Dressing (0.5 mg) served as the positive control (PC). Each sample was tested in triplicate [40].

### 2.8. Migration Rate Determination

NIH/3T3 cells (2 × 10^6^ cells/well) were seeded in 6-well plates. When cells reached 80% confluence, a sterile pipette tip (Ø = 0.1 mm) was used to create a scratch across the center of the monolayer. The culture medium was replaced with a fresh medium containing Ag NP@CHI, Ag NP@CHI@β-1,3-glucan/HA, or CHI@β-1,3-glucan/HA with an Ag concentration of 25 ppm. The scratch-injured cells were incubated until wound closure. Wound healing was observed under an inverted microscope (Leica DMi1, Wetzlar, Germany), and the cell-free area was measured using the software ImageJ v5.0.3 at the leading edges of the scratch at four fixed reference points per well. Measurements were taken immediately after the scratch, after 8 h, and after 24 h [41].

### 2.9. In Vitro Antibacterial Activity

Gram-positive *Staphylococcus aureus* (*S. aureus*, ATCC 6538, Food Industry Research and Development Institute, Hsinchu, Taiwan) and Gram-negative *Escherichia coli* (*E. coli*, BNCC 133264, BeNa Culture Collection, Suzhou, China) were used for the antibacterial assay.

#### 2.9.1. Bacterial Co-Culture Method

The bacteria were amplified to 1 × 10^9^ colony-forming units (CFU)/mL and then diluted with LB medium to a concentration of 1 × 10^4^ CFU/mL. A 1 mL aliquot of bacterial fluid was co-cultured with different Ag NP@CHI at a silver concentration of 100 ppm. Additionally, AgNO_3_, Ag NP@CHI, Ag NP@CHI@β-1,3-glucan/HA, or CHI@β-1,3-glucan/HA with an Ag concentration of 25 ppm was added to 1 mL of bacterial fluid. Bacterial fluid without any sample served as the control. After incubation at 37 °C for 24 h, optical density values at 595 nm were measured using an ELISA microplate reader, and relative bacterial growth inhibition was calculated [42].

#### 2.9.2. Agar Well Diffusion Method

For the agar diffusion assay, 100 μL of bacterial fluid (1 × 10^6^ CFU/mL) was evenly spread on an LB agar plate. Filter papers pre-soaked with AgNO_3_, Ag NP@CHI, Ag NP@CHI@β-1,3-glucan/HA, or CHI@β-1,3-glucan/HA solutions with an Ag concentration of 100 ppm were placed on the plates. Saline-soaked filter papers were used as the control. After 24 h of incubation, the antibacterial effects were evaluated by observing the diameter of the inhibition zones [43].

#### 2.9.3. Plate-Counting Method

To quantify bacterial viability, 1 mL of bacterial fluid (1 × 10^4^ CFU/mL) was co-cultured with AgNO_3_, Ag NP@CHI, Ag NP@CHI@β-1,3-glucan/HA, or CHI@β-1,3-glucan/HA solutions at an Ag concentration of 50 ppm. Bacterial fluid without any sample addition served as the control. After 24 h of incubation at 37 °C, 50 µL of the sample was collected, diluted 50,000-fold with LB medium, and 150 µL of each dilution was spread onto LB agar plates. The plates were incubated overnight at 37 °C, and CFUs were counted [44].

### 2.10. Statistical Analysis

All data are expressed as mean ± SD. One-way ANOVA was performed to determine statistical significance among experimental groups, with a *p*-value < 0.05 considered statistically significant.

## 3. Results and Discussion

### 3.1. Characterization of the Ag NP@CHI

**Figure 1:**
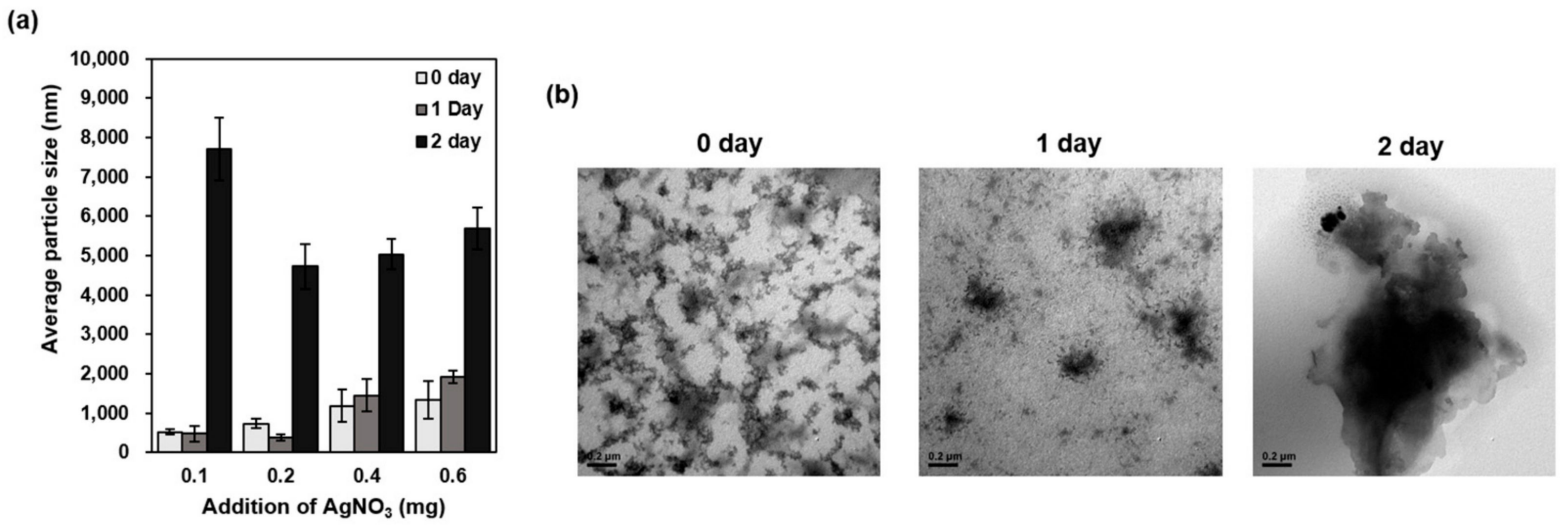
Characteristics of silver nanoparticles-chitosan nanocomposites (Ag NP@CHI) prepared with different contents of silver nitrate (AgNO_3_) in 0.5 mg/mL chitosan solution. (**a**) Z-average particle size of different Ag NP@CHI (*n* = 4). (**b**) Transmission electron microscopy (TEM) images of Ag NP@CHI incubated at 80 °C for 0, 1, and 2 days. Scale bar = 200 nm.

As a multifunctional template, chitosan serves not only as an effective reducing agent in the synthesis of chitosan-silver nanocomposites but also as a stabilizer for these nanocomposites [22,23,24]. During the reduction process of Ag NPs in 0.5 mg/mL chitosan solution, the particle size of Ag NP@CHI was positively correlated with the amount of AgNO_3_ added (Figure 1a) [38]. When Ag NP@CHI was incubated at 80 °C for 1 day, the particle size decreased. This suggests that more Ag^+^ ions attracted to chitosan were reduced to Ag NPs, leading to the aggregation of chitosan molecules and a reduction in particle size (Figure 1b). However, after 2 days of incubation, the particle size increased dramatically due to the aggregation of Ag NP@CHI nanocomposites. Therefore, the particle size of Ag NP@CHI was correlated with both the AgNO_3_ content and incubation time at 80 °C.

**Figure 2:**
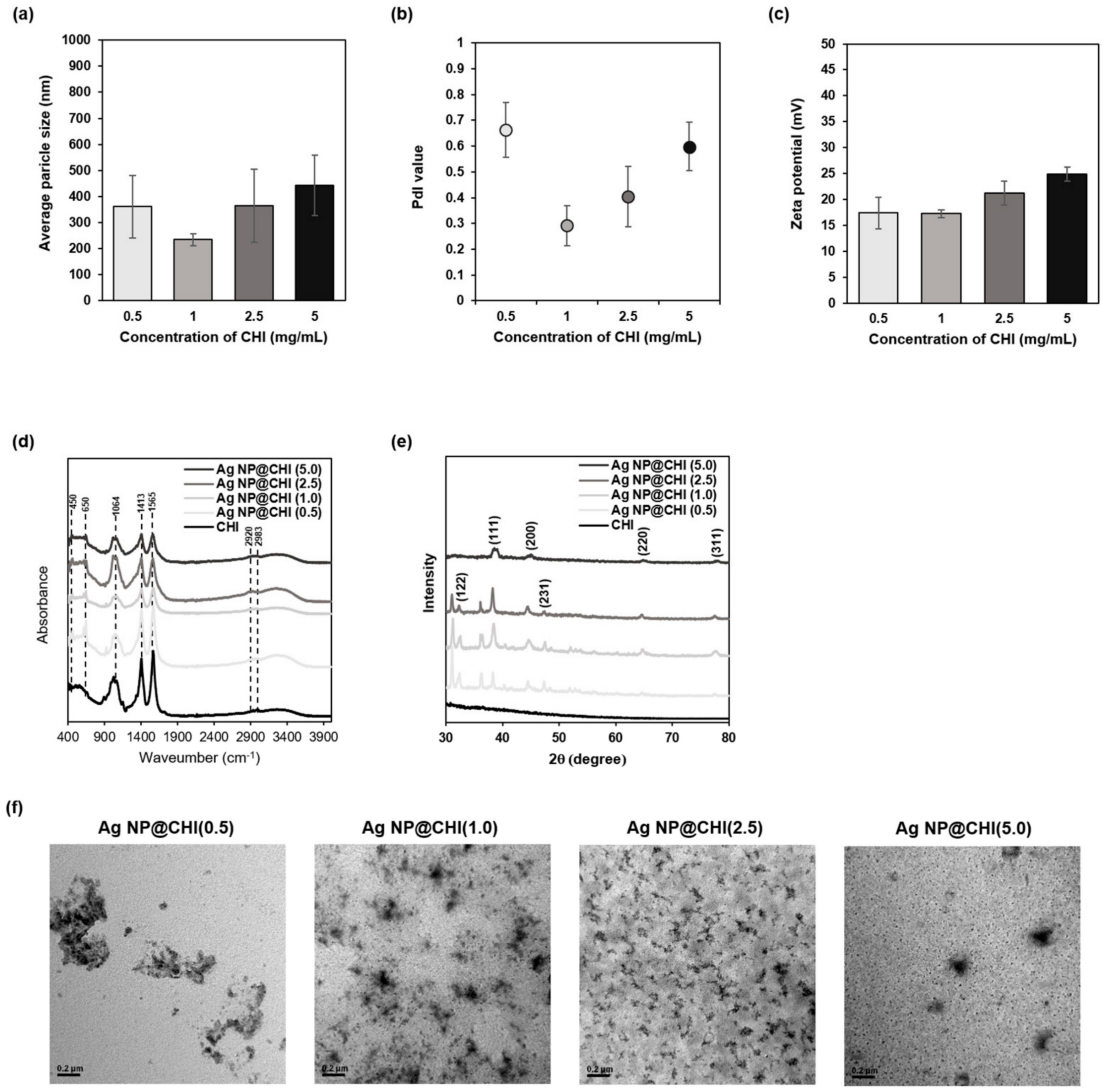
Characteristics of Ag NP@CHI nanocomposites prepared with 0.2 mg AgNO_3_ in different chitosan solutions. (**a**) Z-average particle size of different Ag NP@CHI. (**b**) Polydispersity index (PdI) values of different Ag NP@CHI. (**c**) Zeta potential of different Ag NP@CHI. All data points are represented as mean ± SD (*n* = 4). (**d**) Fourier-transform infrared spectroscopy (FTIR) spectra of chitosan (CHI), Ag NP@CHI (0.5), Ag NP@CHI (1.0), Ag NP@CHI (2.5), and Ag NP@CHI (5.0). (**e**) X-ray diffraction (XRD) diagrams of chitosan (CHI), Ag NP@CHI (0.5), Ag NP@CHI (1.0), Ag NP@CHI (2.5), and Ag NP@CHI (5.0). (**f**) TEM images of Ag NP@CHI (0.5), Ag NP@CHI (1.0), Ag NP@CHI (2.5), and Ag NP@CHI (5.0). Scale bar = 200 nm.

As shown in Figure 2a,b, when the chitosan concentration was 1.0 mg/mL, the smallest particle size and PdI value of Ag NP@CHI were achieved. The surface charge of Ag NP@CHI was positive and increased with the chitosan concentration (Figure 2c). Figure 2d shows the FTIR spectra of chitosan and different Ag NP@CHI nanocomposites. Peaks at 2920 and 2883 cm^−1^ correspond to the stretching vibration of C−H from the methylene and methyl groups of chitosan, respectively. Another peak at 1078 cm^−1^ is attributed to the vibration of C−O−C in chitosan [45]. The amide II peak appeared at 1565 cm^−1^, and the C-CH_3_ deformation vibrational absorption peak at 1413 cm^−1^ indicated that chitosan has a high degree of deacetylation [4]. The successful synthesis of Ag NPs in chitosan was confirmed by the presence of small intensity peaks at 687 and 450 cm^−1^, corresponding to the characteristic Ag−N bond and Ag–O vibration, respectively [46,47], but there was no obvious difference in FTIR spectra of Ag NP@CHI prepared with 0.5, 1.0, 2.5, and 5.0 mg/mL chitosan solutions.

The crystalline structure of the obtained Ag NP@CHI samples was studied using XRD analysis, as shown in Figure 2e. No diffraction peaks were observed in the chitosan spectrum (CHI), but the spectra of Ag NP@CHI prepared with 0.5, 1.0, and 2.5 mg/mL chitosan solutions (Ag NP@CHI (0.5), Ag NP@CHI (1.0), and Ag NP@CHI (2.5)) exhibited six diffraction peaks at 2θ values of 32.38°, 38.28°, 44.48°, 47.36°, 64.67°, and 78.43°, corresponding to the [122], [111], [200], [231], [200], and [311] planes of a standard face-centered cubic Ag NP structure, respectively [48,49]. The spectrum of Ag NP@CHI prepared with a 5.0 mg/mL chitosan solution (Ag NP@CHI (5.0)) displayed four major peaks at 2θ values of 38.28°, 44.48°, 64.67°, and 78.43°, reflecting the [111], [200], [200], and [311] diffractions of the cubic Ag phase, respectively. The diffraction peaks in the spectrum of Ag NP@CH (5.0) were smaller than those in the spectra of Ag NP@CHI (0.5), Ag NP@CHI (1.0), or Ag NP@CHI (2.5), and the two diffraction peaks at 32.38° and 47.36° disappeared. It may be suggested that the high concentration of chitosan (5.0 mg/mL) led to the formation of Ag NPs with a smaller crystalline structure and good dispersion in the chitosan template, causing weakened or absent XRD signals for Ag NPs.

TEM images of different Ag NP@CHI nanocomposites indicated that the 0.5 mg/mL chitosan concentration was effective as a template for synthesizing chitosan-silver nanocomposites, but it was insufficient to stabilize the chitosan-silver nanocomposites in a solution (Figure 2f) and resulted in larger particle size. Increasing the chitosan concentration improved the stability of Ag NP@CHI in solution, and smaller Ag NPs (about 10 nm) were formed and well-dispersed in the chitosan matrix, supporting the XRD results for Ag NP@CHI (5.0). This suggests that the high concentration of chitosan with high viscosity reduced the mobility of Ag^+^ ions, allowing the greatest reduction of Ag NPs to occur in situ without aggregation with nearby Ag^+^ ions or Ag NPs to form larger clusters. These findings explain why larger PdI values for Ag NP@CHI (2.5) and Ag NP@CHI (5.0) were observed in the Zetasizer measurements.

### 3.2. Antibacterial Activity of Ag NP@CHI

**Figure 3:**
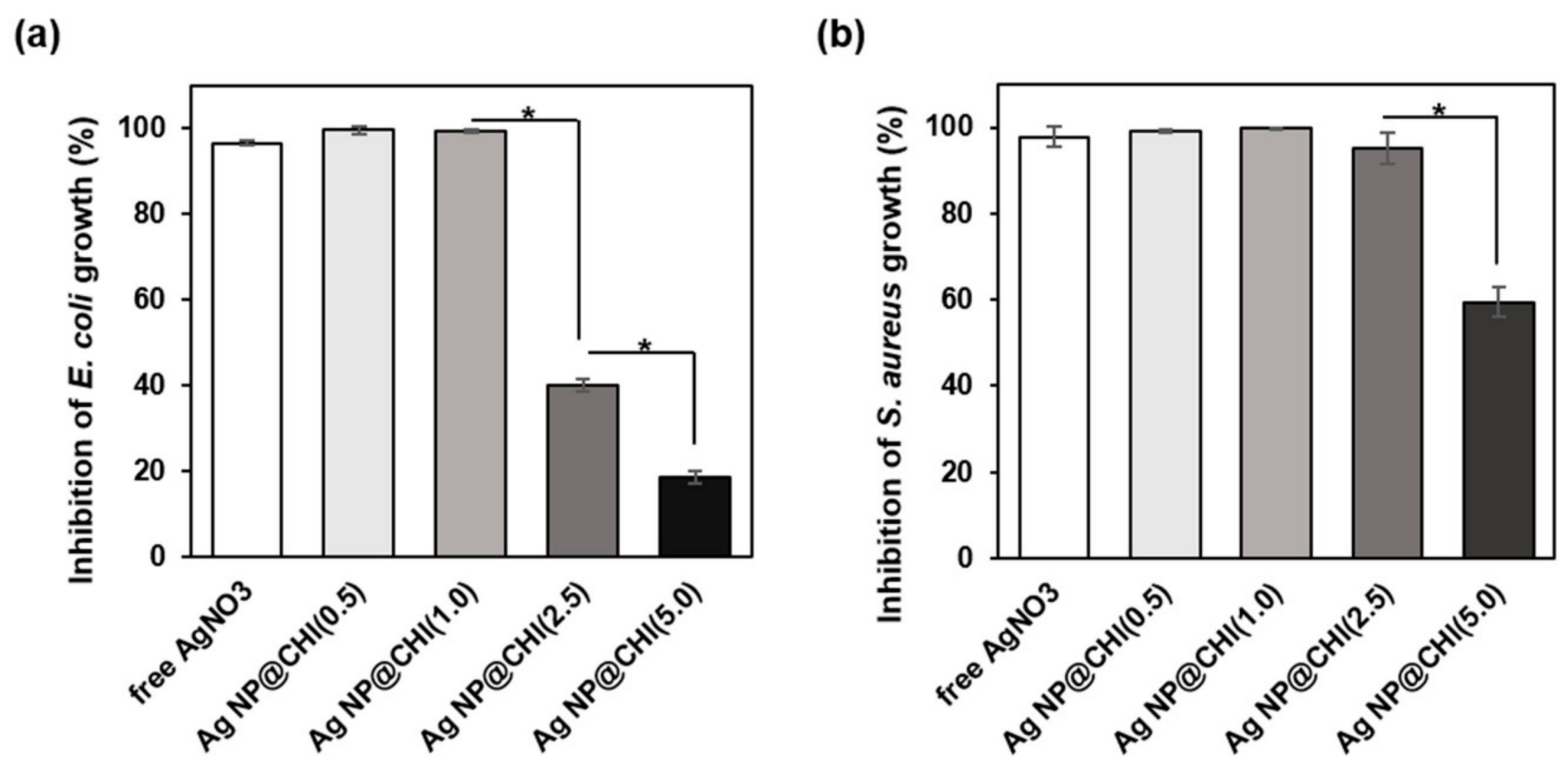
Inhibition of *Escherichia coli* (**a**) and *Staphylococcus aureus* (**b**) growth by silver nitrate (AgNO_3_) and different Ag NP@CHI nanocomposites at Ag concentration of 100 ppm. *n* = 4; * *p* < 0.05.

Silver has long been used as a relatively harmless antibacterial material and disinfectant. Due to their nanoscale size and large surface area-to-volume ratio, Ag NPs can increase the permeability of cell membranes, generate reactive oxygen species, and interrupt DNA replication by releasing silver ions, thereby causing cell death [50]. As shown in Figure 3a, the Ag NP@CHI prepared with chitosan concentrations of 0.5 and 1.0 mg/mL (Ag NP@CHI (0.5) and Ag NP@CHI (1.0)) demonstrated inhibition of *E. coli* growth comparable to free AgNO_3_ solution at an Ag concentration of 100 ppm. However, the inhibition efficacy decreased significantly with an increase in chitosan concentration. It is suggested that high chitosan concentrations provided sufficient stability to the Ag NPs in the chitosan matrix but limited the direct interaction of Ag NPs with *E. coli*, resulting in antibacterial effects derived primarily from chitosan. It has been indicated that the size of chitosan nanoparticles is negatively correlated with antimicrobial activity [51,52]. Therefore, Ag NP@CHI (2.5) showed greater growth inhibition of *E. coli* than Ag NP@CHI (5.0). Similar results were observed for *S. aureus*, but the growth inhibition of Ag NP@CHI (2.5) and Ag NP@CHI (5.0) on *S. aureus* was higher than that on *E. coli*. Chitosan generally exhibits stronger effects on Gram-positive bacteria (*S. aureus*) than on Gram-negative bacteria (*E. coli*) [53,54,55], confirming that the antibacterial activity of Ag NP@CHI (2.5) and Ag NP@CHI (5.0) mainly contributed from chitosan not Ag NPs. Since Ag NP@CHI (1.0) had the smallest particle size and good antibacterial activity against both *E. coli* and *S. aureus*, it was selected for mixing with β-1,3-glucan/HA for further study.

### 3.3. Characterization of Ag NP@CHI@β-1,3-Glucan/HA

**Figure 4:**
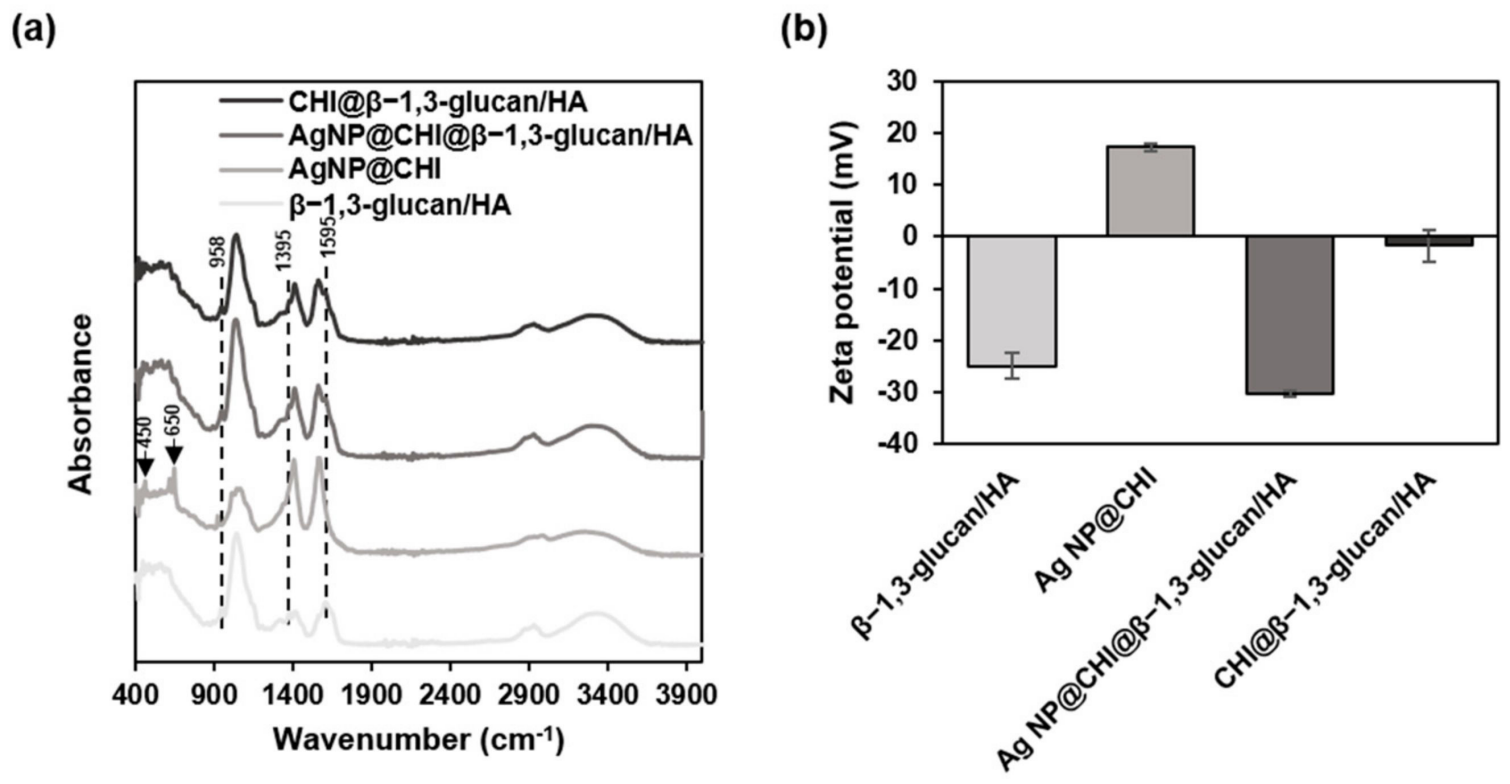
Characteristics of β-1,3-glucan/hyaluronic acid (β-1,3-glucan/HA), Ag NP@CHI, Ag NP@CHI@β-1,3-glucan/HA, and CHI@β-1,3-glucan/HA. (**a**) FTIR spectra of β-1,3-glucan/HA, Ag NP@CHI, Ag NP@CHI@β-1,3-glucan/HA, and CHI@β-1,3-glucan/HA. (**b**) Zeta potential of β-1,3-glucan/HA, Ag NP@CHI, Ag NP@CHI@β-1,3-glucan/HA, and CHI@β-1,3-glucan/HA (*n* = 4).

Figure 4a presents the FTIR spectra of β-1,3-glucan/HA, Ag NP@CHI, Ag NP@CHI@β-1,3-glucan/HA, and CHI@β-1,3-glucan/HA. When Ag NP@CHI was mixed with β-1,3-glucan/HA, the peaks at 450 and 650 cm^−1^, attributed to the Ag−N bond and Ag–O vibration of Ag NP@CHI, respectively, disappeared, likely shielded by β-1,3-glucan/HA, and the characteristic peaks of β-1,3-glucan/HA appeared. For example, the band at 1595 cm^−1^ was assigned to the asymmetric stretching mode of the planar carboxyl groups, while the band at 946 cm^−1^ was typical of carbohydrates in β-1,3-glucan/HA. Additionally, the peak at 1395 cm^−1^ indicated *v_s_* (CO) (of COO−) in HA [56].

β-1,3-glucan is a glucose polymer that is slightly negatively charged due to phosphate residues [57], while HA is a negatively charged polysaccharide due to the carboxylate groups in its structure [58]. Therefore, when positively charged Ag NP@CHI was mixed with β-1,3-glucan/HA to form Ag NP@CHI@β-1,3-glucan/HA, the positive charge of Ag NP@CHI was neutralized, and the charge of Ag NP@CHI@β-1,3-glucan/HA became negative (Figure 4b). A similar result was observed for CHI@β-1,3-glucan/HA.

### 3.4. Hemocompatibility and CBT of Ag NP@CHI and Ag NP@CHI@β-1,3-Glucan/HA

**Figure 5:**
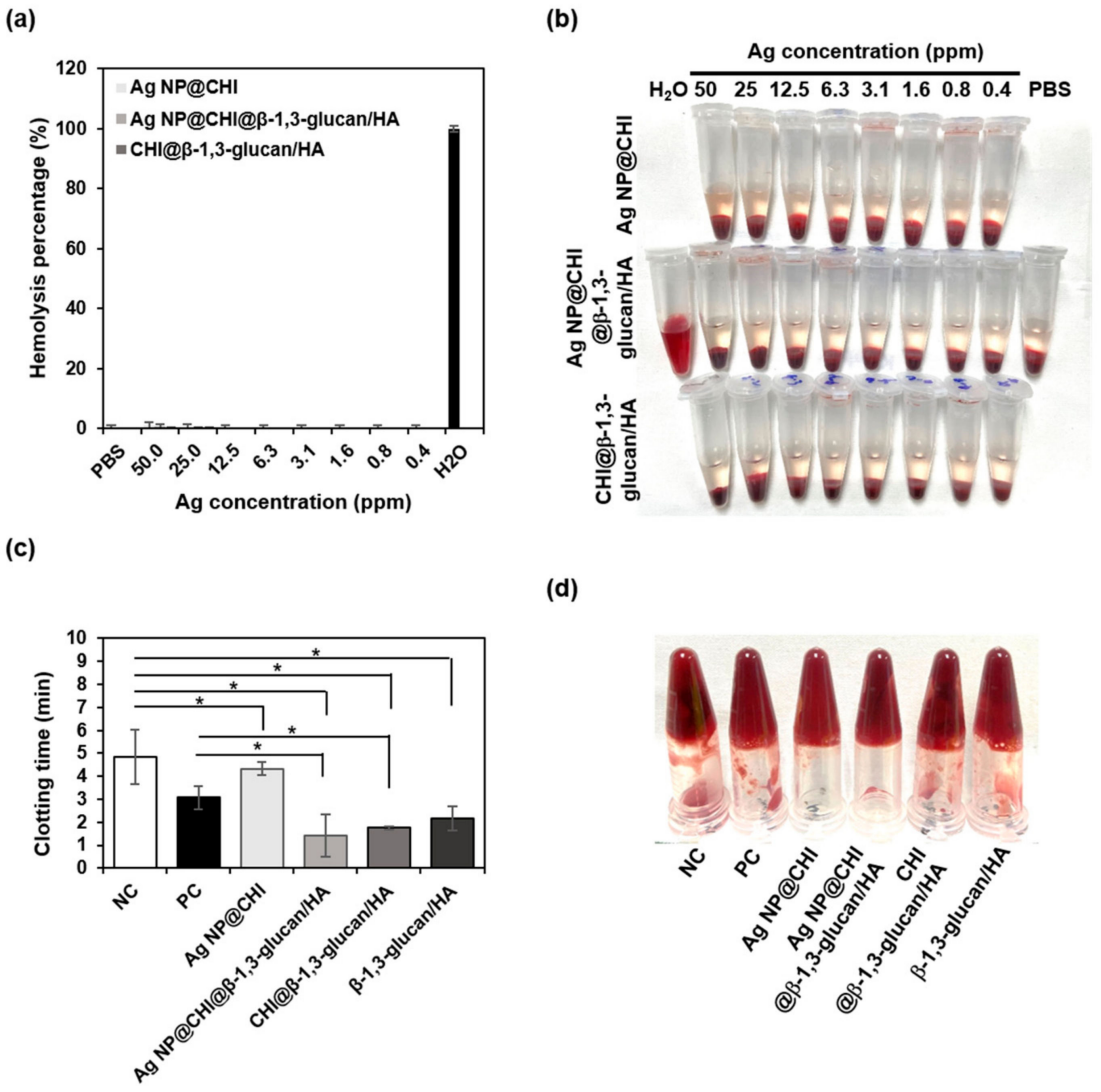
(**a**) Relative hemolysis induced by Ag NP@CHI, Ag NP@CHI@β-1,3-glucan/HA, and CHI@β-1,3-glucan/HA at different silver concentrations. Phosphate-buffered saline (PBS) and H_2_O were used as negative and positive controls, respectively (*n* = 3). (**b**) Images of blood mixtures with Ag NP@CHI, Ag NP@CHI@β-1,3-glucan/HA, and CHI@β-1,3-glucan/HA at different silver concentrations; PBS, and H_2_O after red blood cells were pelleted. Clotting blood time (CBT) (**c**) and blood clotting (**d**) after adding different hemostatic materials into whole blood (*n* = 3). The same mass (5 mg) of different materials was used. NC: CaCl_2_ only; PC: HealiAid^®^ Collagen Wound Dressing. * *p* < 0.05.

Hemocompatible wound dressings are designed to minimize damage to red blood cells and promote hemostasis, the first step in wound healing [59,60]. Here, not only Ag NP@CHI but also Ag NP@CHI@β-1,3-glucan/HA and CHI@β-1,3-glucan/HA demonstrated hemolytic potential as low as PBS, even at high Ag concentrations (50 ppm) (Figure 5a,b), indicating the safety and good blood compatibility of the prepared composites.

An ideal wound dressing should not only be biocompatible and exhibit high antibacterial activity but also promote blood coagulation at the wound site. Compared to CaCl_2_ only, the CBT of Ag NP@CHI@β-1,3-glucan/HA, CHI@β-1,3-glucan/HA, and β-1,3-glucan/HA was significantly reduced (Figure 5c,d). β-1,3-glucan can enhance plasma clotting by activating factor XII, binding with fibrinogen, and increasing the local concentration of clotting factors via steric exclusion [61]. Thus, incorporating β-1,3-glucan improved blood coagulation. Moreover, the chitosan in Ag NP@CHI@β-1,3-glucan/HA and CHI@β-1,3-glucan/HA could attract negatively charged blood cells and platelets, facilitating agglutination formation [62] and reducing CBT. The CBT of Ag NP@CHI@β-1,3-glucan/HA was significantly lower than the HealiAid^®^, indicating its excellent promotion of the coagulation effect.

### 3.5. Cytotoxicity and Cell Migration Induced by Ag NP@CHI and Ag NP@CHI@β-1,3-Glucan/HA

**Figure 6:**
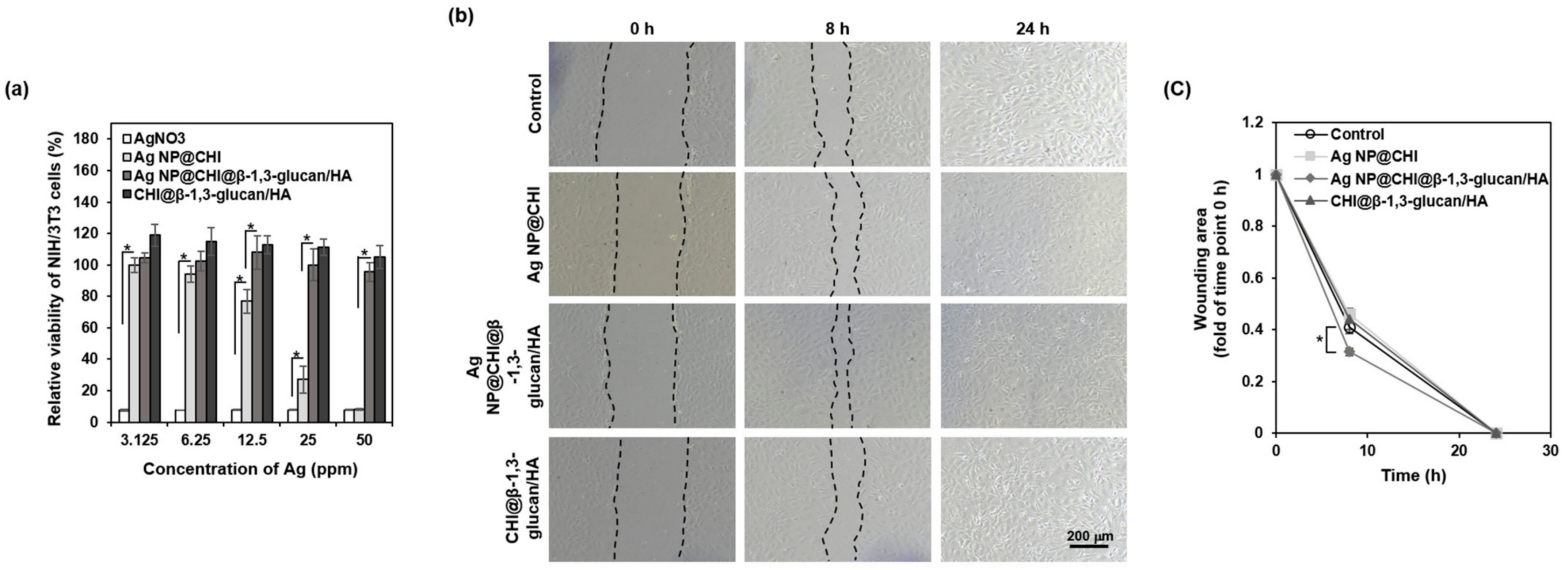
(**a**) The relative viability of NIH/3T3 cells after treatment with AgNO_3_, Ag NP@CHI, Ag NP@CHI@β-1,3-glucan/HA, and CHI@β-1,3-glucan/HA at different silver concentrations for 24 h. (**b**) Representative bright-field images of NIH/3T3 cells migration treated with Ag NP@CHI, Ag NP@CHI@β-1,3-glucan/ HA, and CHI@β-1,3-glucan/HA at an Ag concentration of 25 ppm. The bar is 200 µm. (**c**) Wound closure expressed as the remaining area uncovered by the cells. The scratch area at time point 0 h was set to 1 (*n* = 3; * *p* < 0.05).

AgNO_3_ can inhibit DNA synthesis and is associated with a significant loss of cellular proteins, causing notable inhibition of normal cell proliferation and leading to Ag-dependent cell loss. Moreover, ionic silver induces concentration- and time-dependent depletion of intracellular ATP content, impairing the cellular energy charge before human dermal fibroblast death [63]. As shown in Figure 6a, AgNO_3_ caused serious cell damage even when the Ag concentration was as low as 3.125 ppm. However, after the formation of Ag NP@CHI composites, cytotoxicity was significantly reduced but remained concentration-dependent. This indicates that the toxicity of ionic silver can be reduced by limiting its release from Ag NP@CHI. Due to the high biocompatibility of HA and β-1,3-glucan, the prepared Ag NP@CHI@-1,3-glucan/HA exhibits low cytotoxicity even at an Ag concentration of 50 ppm.

Figure 6b,c illustrate the results of the in vitro cell migration test. After an 8 h incubation period, approximately 59% of the damaged area healed due to the migration and proliferation of NIH/3T3 cells into the scratched region (control group). Compared to the control group, cells treated with Ag NP@CHI displayed approximately 54.0% of the damaged area healed, indicating the toxicity of Ag NP@CHI with Ag concentration of 25 ppm repressed the cell migration and proliferation. A significant difference in wound contraction was observed in the Ag NP@CHI@β-1,3-glucan/HA treated group (68.6%) compared with the control group, suggesting the promotion of cell migration was contributed from β-1,3-glucan and HA [29,30,37].

### 3.6. In Vitro Antibacterial Activity of Ag NP@CHI and Ag NP@CHI@β-1,3-Glucan/HA

**Figure 7:**
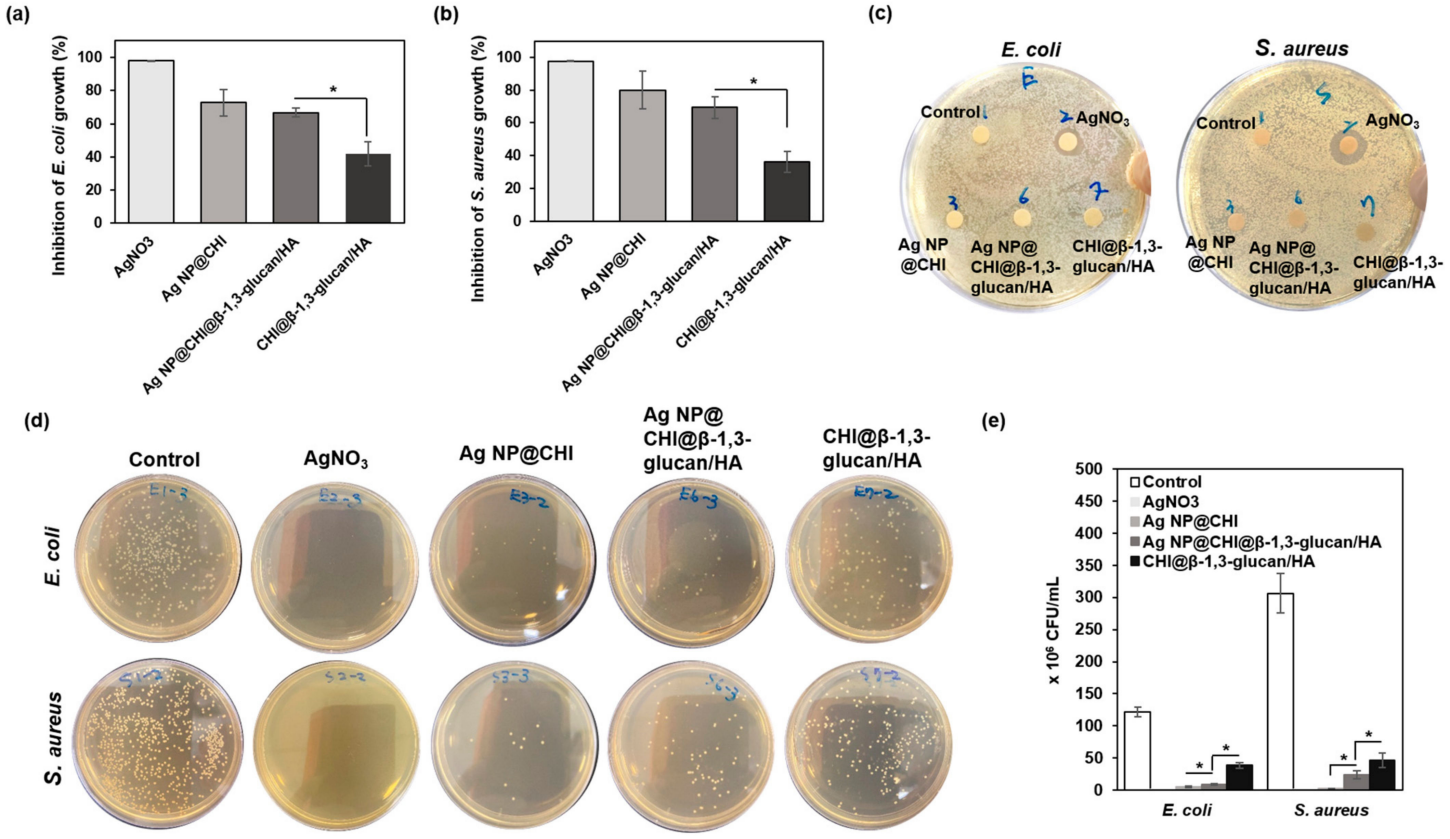
The antibacterial activity of AgNO_3_, Ag NP@CHI, Ag NP@CHI@β-1,3-glucan/HA, and CHI@β-1,3-glucan/HA with Ag concentration of 25 ppm against *E. coli* (**a**) and *S. aureus* (**b**) (*n* = 4). (**c**) Photos of the inhibition zones of AgNO_3_, Ag NP@CHI, Ag NP@CHI@β-1,3-glucan/HA, and CHI@β-1,3-glucan/HA with Ag concentration of 100 ppm incubated with *E. coli* and *S. aureus* for 24 h. Photos (**d**) and quantitative analysis (**e**) of *E. coli* and *S. aureus* bacterial colonies incubated with AgNO_3_, Ag NP@CHI, Ag NP@CHI@-1,3-glucan/HA, and CHI@-1,3-glucan/HA at an Ag concentration of 50 ppm for 24 h (*n* = 3; * *p* < 0.05).

As shown in Figure 7a, Ag NP@CHI with an Ag concentration of 25 ppm displayed 73% inhibition of *E. coli* growth, which was lower than AgNO_3_ (98%). However, mixing Ag NP@CHI with HA and β-1,3-glucan and then incubating it with *E. coli* reduced the inhibition of *E. coli* growth to 66%, suggesting that the diffusion of ionic silver was limited by the β-1,3-glucan/HA composite. Since chitosan and β-glucans possess potential antibacterial activity against various Gram-positive and Gram-negative bacteria [9,36], the CHI@β-1,3-glucan/HA exhibited 42% suppression of *E. coli* growth. Similar results were observed for *S. aureus* growth inhibition (Figure 7b), where AgNO_3_, Ag NP@CHI, Ag NP@CHI@β-1,3-glucan/HA, and CHI@β-1,3-glucan/HA showed suppression rates of 98%, 80%, 69%, and 36%, respectively.

Figure 7c shows the antibacterial activity of AgNO_3_, Ag NP@CHI, Ag NP@CHI@β-1,3-glucan/HA, and CHI@β-1,3-glucan/HA against *E. coli* and *S. aureus*. A clear inhibition zone was observed for AgNO_3_, while a slight outline surrounding the filter paper pre-soaked with Ag NP@CHI was noted. However, no inhibition zone was observed for Ag NP@CHI@β-1,3-glucan/HA and CHI@β-1,3-glucan/HA, differing from the results in Figure 7a,b. This suggests that the diffusion of ionic silver from Ag NP@CHI@β-1,3-glucan/HA and CHI@β-1,3-glucan/HA was restricted under these test conditions, resulting in reduced antibacterial activity.

Colony-forming unit (CFU) measurements indicated that AgNO_3_, Ag NP@CHI, Ag NP@CHI@β-1,3-glucan/HA, and CHI@β-1,3-glucan/HA exhibited antibacterial effects against *E. coli* and *S. aureus* (Figure 7d,e). However, the antibacterial activity followed the order AgNO_3_ > Ag NP@CHI > Ag NP@CHI@β-1,3-glucan/HA, suggesting that the chitosan template and β-1,3-glucan/HA restricted ionic silver release and diffusion, thereby reducing the antibacterial effect on *E. coli* and *S. aureus*. Interestingly, the antibacterial effect of Ag NP@CHI, Ag NP@CHI@β-1,3-glucan/HA, or CHI@β-1,3-glucan/HA against *S. aureus* was higher than that against *E. coli*. Some references indicate that Ag NPs are more effective against Gram-negative bacteria than Gram-positive bacteria due to the ease of attachment of Ag NPs to the thin peptidoglycan layer and thick LPS layer of Gram-negative bacterial cell walls [64,65,66]. Therefore, the antibacterial effect of Ag NP@CHI, Ag NP@CHI@β-1,3-glucan/HA, or CHI@β-1,3-glucan/HA may primarily originate from chitosan, with a secondary contribution from the slowly released ionic silver. Chitosan and β-1,3-glucan in the composite could initially interact with the negatively charged outer or inner membrane of bacteria through electrostatic interactions, or damage membrane integrity through interactions with phospholipids and proteins, altering the permeability of the bacterial cell membrane. Subsequently, the released silver ions could further damage the cell wall and cytoplasmic membrane, denature bacterial ribosomes, interrupt adenosine triphosphate (ATP) production, interfere with DNA replication, and even cause membrane perforation, leading to the release of organelles from the cell, directly killing the bacteria.

## 4. Conclusions

In this study, chitosan was used as a reducing and capping agent in the synthesis of various chitosan-silver nanocomposites by adjusting the chitosan concentration and incubation time at 80 °C. A high concentration of chitosan resulted in the formation of Ag NPs with a smaller crystalline structure and good dispersion within the chitosan matrix. Moreover, chitosan could reduce the direct toxicity of Ag NPs on normal cells but also lower their antibacterial potency at low ionic silver concentrations. Incorporating Ag NP@CHI with β-1,3-glucan/HA significantly improved the biocompatibility and hemocompatibility of Ag NP@CHI, with a slight reduction in antibacterial efficiency due to the restricted release and diffusion of ionic silver from Ag NP@CHI@β-1,3-glucan/HA. Furthermore, the addition of β-1,3-glucan/HA enhanced the ability of Ag NP@CHI@β-1,3-glucan/HA to promote blood coagulation and wound healing significantly. Owing to its high biocompatibility, hemocompatibility, promotion of blood coagulation and wound healing, and antibacterial efficiency, the prepared Ag NP@CHI@β-1,3-glucan/HA shows potential as a wound healing composite in the future.

## Data Availability

The data presented in this study are available on request from the corresponding author.
